# Evidence for Placental HPV Infection in Both HIV Positive and Negative Women

**DOI:** 10.4236/jct.2015.615140

**Published:** 2015-12-25

**Authors:** Chrispin Chisanga, Dawn Eggert, Charles D. Mitchell, Charles Wood, Peter C. Angeletti

**Affiliations:** 1 Nebraska Center for Virology, School of Biological Sciences, University of Nebraska-Lincoln, Lincoln, NE, USA; 2 University of Miami Miller School of Medicine, Miami, FL, USA

**Keywords:** Human Papillomaviruses (HPVs), HPV Genotypes

## Abstract

Human papillomaviruses (HPVs) have previously been reported to infect epithelial trophoblast cells of the placenta. To investigate this possibility, 200 placental samples from Zambian women were separated into HIV+ and HIV− groups and tested for HPV by redundant primer PCR, using GP5+/GP6+ and CPI/CPII primer sets. Three HPV genotypes (HPV6, 16 and 90) were detected in placental samples. Whereas, 20 different HPV genotypes were detected in vaginal sampling of the same patients, suggesting that compartment specific sub-populations of HPV may exist. The incidence of HPV16 in placental samples was almost 2-fold greater in HIV+ women compared to HIV− (p = 0.0241). HPV16 L1 expression, detected by immunochemistry, was significantly higher in HIV+ than HIV− samples (p = 0.0231). HPV16 DNA was detected in the nuclei of trophoblast cells by *in situ* hybridization. Overall, these results suggest that HPVs infect the placenta and that HIV significantly influences these infections.

## 1. Introduction

Human papillomaviruses are small, non-enveloped double-stranded DNA viruses that infect squamous epithelial cells via micro-abrasions [1]. To date, approximately 200 different genotypes of HPVs have been identified [2] [3]. Two-thirds of these infect cutaneous membranes and one-third infects mucosal membranes. Based on their ability to cause malignant carcinomas, the HPVs that target the mucosa can be categorized into the high-risk (HPV16, 18, 31) and the low-risk types (HPV6, 11) [4]. Infection with the low-risk HPVs usually results in benign epithelial warts, while infection with the High-risk HPVs can lead to anogenital malignancies, among these, cervical cancer [5].

Recent studies have suggested that HPVs can infect the placenta. Transplacental transmission of human papillomaviruses (HPVs) has been documented, with type-specific HPV concordance occurring between the mother, the placenta and the newborn or the mother and cord blood [6]. Another recent study showed that HPV DNA could be detected in 5% of neonates born to healthy women and that the HPV DNA is associated with detection of the same HPVs in mothers in all trimesters of pregnancy. Evidence of maternal-to-fetal transmission of HPV across the placental membrane is not without controversy [7]. This is because placental contamination with cervical cells from an infected birth canal cannot be ruled out as a source of the HPV DNA. However, placental samples obtained from women undergoing trans-abdominal chorionic villous sampling have also revealed the presence of HPV16 and HPV62 in the placenta [8]. Other studies have confirmed detection of HPV type18 in both placental and cervical tissues of pregnant women, with the occurrence of placental HPV infection being unrelated to the mode of child delivery [9]. Further evidence by *in situ* hybridization has shown that HPV DNA can be localized in placental trophoblasts [10].

The trophoblast 3A cell line has been reported to support HPV16 and 31 replication, though the permissiveness of primary trophoblasts to HPV infection *in vivo* remains unclear [11] [12]. Besides mediating nutrient and gas exchange between the fetus and mother, the fetal trophoblast cells are in direct contact with the maternal tissues and play a crucial role in the process of placentation [13]. Based on this intimate contact and communication between the maternal and fetal sides of the placenta, it is thought that infection with HPV may result in death of placental trophoblasts, or malfunction in recognition of endometrial cells or even malignancy. Changes may consequently disrupt the integrity of the trophoblast layer and cause spontaneous abortions or preterm delivery [13] [14]. Together, these studies motivated us to investigate placental HPV, particularly in the context of HIV infection.

Human papillomavirus (HPV) infections are more abundant in human HIV-positive individuals [15]. HIV-positive women have a high prevalence of HPV-induced dysplasias of the cervix [16]–[18]. Similarly, a study conducted by Ahdieh and colleagues showed that HIV-positive women were 1.8, 2.1 and 2.7 times more likely to harbor high-, intermediate-, and low-risk HPV infections, respectively, than HIV-negative women. The persistence of HPV lesions was approximately two times longer in women with a CD4 cell count less than 200 cells/μl compared to those with greater than 500 cells/μl [19].

The risk for acquisition of HPV is directly related to the number of sexual partners. This is in agreement with other findings that HIV-positive individuals tend to have a higher prevalence of anogenital HPV infections [17] [20], with a lower CD4+ count, which is predictive of anogenital intraepithelial neoplasia. The importance of cell-mediated immunity in the control of HPV infection has been evidenced by studies that have documented an increased prevalence and progression of HPV infections in the immunosuppressed [21] [22]. Multiple recurrences of cervical HPV infections occur in HIV infected patients, and anti-retroviral drugs do not appear to thoroughly suppress these relapses [22] [23]. HIV attenuates the systemic immune response against HPV via its effect on CD4+ cells and regulation of immune responses to different types of antigens. A low number of circulating HPV specific memory cells is thought to make the HPV-specific immunity defective and promote disease progression [17].

The derepression of high-risk HPV replication by HIV has been reported elsewhere [17] [21] [22]. However, there is still relatively little known about the role of HIV in HPV pathogenesis in the placental compartments. In the present study, we chose to investigate HPV genotype distributions in the placental compartment as a function of HIV status. We found evidence of 3 HPV genotypes in placenta (type 6, 16 and 90), whereas, vaginal sampling of the same patient population recovered 20 different HPV genotypes [24]. In our previous study, we found that HIV infection was associated with higher incidence of HPV18 in the vaginal compartment. In the present study, we find that HIV-positive patients are about 2-fold more likely to test positive for HPV16 in the placental compartment then those who are HIV-negative, supporting the concept that HPV infections placenta is evidence of HPV16 L1 major capsid protein expression in placental samples from either HIV positive or negative women. Furthermore, we performed *in situ* hybridization, which showed the presence of HPV16 DNA in placental trophoblast cells.

## 2. Materials and Methods

### Study Participants

Between March 1998 and October 2004, 3161 pregnant women who were admitted to the labor ward at the University Teaching Hospital (UTH) in Lusaka, Zambia, were recruited for a cohort study to investigate HIV infection. This study was under institutional IRB approval at the University of Nebraska-Lincoln and the University of Zambia. Women were enrolled into the study with written consent. Placentas from 213 women were selected randomly collected for further study.

### 2.1. Sample Collection

**Figure 1:**
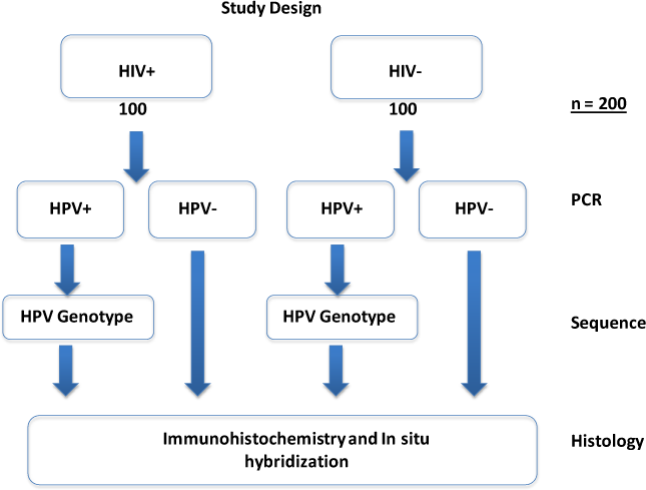
Placental HPV infection study design. A cohort of 200 HIV+ and HIV− paraffin embedded placental tissues was used in the study. Initially, genomic DNA was extracted and PCR amplified using GP5+/GP6+ and CPI/II primers. Beta (*β*)-actin primers were also used as controls. The expected 150 bp PCR product was cloned into the pGEM-T Easy Vector System I followed by direct sequencing. Genotyping was achieved by aligning the sequences and blasting against the NCBI data base. Histological analysis of HPV16 in the tissues was done using immunohistochemistry (IHC).

All tissues were obtained, under consent, from HIV-negative and HIV-positive Zambian women at the time of delivery. The samples were fixed in formalin and embedded in paraffin prior to being shipped to Nebraska Center for Virology (NCV) at University of Nebraska-Lincoln (UNL). To be included in the study, the placental tissue sample of the index patient had to have consented to an HIV test. A total of 200 paraffin embedded placental tissues were assayed in this study: 100 HIV-positive and 100 HIV-negative (Figure 1).

### 2.2. Genomic DNA Extraction from Paraffin Embedded Tissues

Genomic DNA was extracted from 200 paraffin embedded placental tissue samples using the Phenol-Chloroform extraction protocol as described by Pikor *et al.* [25]. Each of the paraffin embedded placental tissues was micro-sectioned and treated with 800 μl of xylene, to dissolve the paraffin wax from the tissues, followed by rehydration using a series of 800 μl ethanol (100%, 70% and 50%) washes. Tissue digestion was achieved by addition of 20 μl (20 mg/ml) of proteinase K, twice a day for three days while incubating the samples at 56°C in a heating block (Incublock™, Denville Scientific Inc.). This ensured that the tissue dissolved completely. The DNA was cleaned up by the phenol-chloroform extraction method after which it was treated with 100 μg/ml of RNase A to remove any contaminating cellular RNA. Finally, the purified DNA was eluted in 50 μl of nuclease free water and quantified by the Nano Drop Spectrophotometer (*ND*-1000).

### 2.3. HPV Amplification and Beta (*β*)-Actin

The DNA extracted from placental tissue samples were amplified using regular PCR. Two redundant primers, (**GP5+**: 5′-TTT GTT ACT GTG GTA GAT ACT AC-3′ and **GP6+**: 5′-GAA AAA TAA ATG TAA ATC ATA TTC-3′) that amplify 150 bp of the L1 region (nt 6624-6746) of the HPV genome was used to detect HPV (24) in the samples. Another pair of redundant primers (**CPI**: 5′-TTA TCWTAT GCC CAY TGT ACC AT-3′ and **CPII**: 5′-ATG TTA ATW SAG CCW CCA AAA TT-3′) which are targeted to the E1 region (nt 1777–1964) and amplify 188 bp of the HPV genome was also used to detect HPV in the samples. We also used *β*-Actin primers (Forward Primer: 5′-GCC ATG TAC GTT GCT ATC C-3′ and Reverse Primer: 5′CCG CGC TCG GTG AGG ATC-3′). The use of these sets of primers on our samples and the simultaneous detection of HPV with both sets of primers provided a robust set of results for our analysis. The thermal cycler model, TECHNE, TC-412, was used for amplification. The parameters for denaturation, hybridization and extension were as follows: 94°C for 1 minute, followed by 30 cycles of 95°C for 30 seconds, 55°C for 1 minute, 72°C for 10 minutes and final hold at 4°C. The positive control constituted the HPV16 Plasmid DNA (pEF399), whereas the negative control was nuclease free water. To determine the presence or absence of HPV fragments and of *β*-actin amplified from the oligonucleotides, 30 μl of the PCR product from each sample was pre-mixed with 1.5 μl 6× loading dye and separated by gel electrophoresis on 2% (w/v) agarose gel, in 1× TAE buffer. At the end of electrophoresis, the gel was stained with 0.3% ethidium bromide (0.1 mg/μl solution) for 30 minutes and visualization of the DNA fragments was performed under ultraviolet light.

### 2.4. Cloning and Sequencing of PCR Products

Following PCR amplification of genomic DNA, the 150 bp amplicon was excised and purified from the 2% (w/v) agarose gel using the Qiaquick gel extraction kit (Qiagen) after which 1 μl of the PCR product was cloned into the pGEMT-Easy Vector system I (Promega Corporation. WI, USA) and incubated at 4°C overnight. All the plasmid DNA samples containing the 150 bp insert were analyzed by Direct Sequencing using the ABI Prism Big Dye Terminator v3.1 Cycle Sequencing using conditions prescribed by the manufacturer. The HPV genotypes were determined by comparison with the NCBI GenBank database (Figure 1).

### 2.5. Immunohistochemistry (IHC) Staining

To identify trophoblast cells, we stained tissue sections with a monoclonal antibody against hydroxyl-delta-5-steroid dehydrogenase (HSD3B1) as recommended by Mao *et al.* [26]. The slides containing the sectioned tissues were baked for an hour at 60°C in an incubator and allowed to cool for 30 minutes at room temperature before rehydrating with 5 minute incubations in Xylene 1 and 2, Absolute alcohol 1 and 2, 85% Alcohol and 75% Alcohol. Endogenous peroxidase in the tissues was blocked for 20 minutes with 2 mL 30% Hydrogen peroxide per 200 mL methanol, followed by rinsing in distilled water three times for three minutes. Next, the slides were treated in 0.02% Sodium citrate (v/v) and cooked for 20 minutes at 95°C to unmask the epitopes, after which they were cooled for 20 minutes at room temperature, while still immersed in the sodium citrate solution. This was followed by a rinse in Phosphate Buffered Saline (PBS) for 5 minutes and the tissue sections were rimmed with a pap pen, carefully making sure that the slides did not dry out. Blocking was achieved by incubating the slides in 150 μL 10% Normal Goat Serum (10% NGS in PBS) for 30 minutes in a humidity chamber containing a little water. Next, the blocking solution was flicked off the slides and 150 μL of the Monoclonal anti-HSD3B1 produced in mouse [SIGMA-ALDRICH] was added at a dilution of 1:2000. The primary antibody was, however, not added to the negative control slide. This was followed by an hour of incubation in a humidity chamber. Next, the slides were rinsed in PBS; three changes, three minutes each followed by addition of three drops of anti-mouse DAKO Envision + Horseradish Peroxidase (HRP) labeled Polymer (REF: K4001) and incubation in a humidity chamber for 30 minutes at room temperature. The slides were again rinsed in PBS; three changes; three minutes each. Using one slide, 200 μL 2,3-Diaminobenzidamine (DAB) solution (1 drop of DAKO DAB+ Chromogen −REF: K3468 and 1 mL per 1 mL of DAKO DAB+ substrate buffer-REF: K3468) was added and stain development was observed under the microscope, taking note of the time for optimal intensity. The rest of the slides were then developed using the same time that the DAB solution on the trial slide took to develop to the desired intensity. The slides were then washed in 200 mL of distilled water using two changes for five minutes each, followed by dipping them in undiluted hematoxylin for 20 seconds. The hematoxylin was washed off by letting the slides sit in running tap water for two minutes and then dipping them in ammonia water (500 μL ammonia + 1000 mL of water) for 12 seconds, followed by dehydration sequentially as follows: 70% Alcohol; 85% Alcohol; 100% Alcohol; 100% Alcohol for five minutes each and two changes of Xylene for five minutes each. Finally, cytoseal was used to coverslip the slides, leaving them overnight to dry before microscopic examination the following day.

Using the same IHC protocol we used an anti-V5 L1 monoclonal antibody (mAb) to probe for the HPV16 L1 protein in the placental trophoblasts. The mouse monoclonal anti-V5L1 mAb is a type-specific neutralizing antibody which had been previously raised against human papillomavirus (HPV) type 16 L1 and was able to block more than 75% infectivity [27]. In our study, a 1:250 dilution of the mouse monoclonal anti-L1 gave the best staining results for the L1 protein.

### 2.6. HPV16 L1 Quantification

For this purpose, two slides of each sample were quantified and averaged. Quantification of the HPV16 L1 protein levels in both HIV-negative and HIV-positive tissue sections was performed using Image-Pro Version 9.0. The average HPV16 L1 protein expression in each sample was normalized to the tissue area. We also quantified HPV16 L1 expression in the HPV negative tissues to determine the baseline. Statistical analysis to compare how the relative HPV16 L1 expression varied among groups was performed using the Kruskal-Wallis method in GraphPad Prism 5. Further comparisons to determine differences, if any, in the relative HPV16 L1 signal between the HIV−/HPV16+ and HIV+/HPV16+ groups were compared using the Mann-Whitney test.

### 2.7. HPV16 DNA *In-Situ* Hybridization

One μg of HPV16 DNA was linearized with BamHI restriction enzyme and denatured by boiling. DNA was then labeled with dUTP-biotin using the High-Prime biotin labeling kit (Roche). Tissue mounted on slides was baked at 65°C for 1 hr in an oven. Slides were then extracted with xylene and rehydrated with successively lower concentrations of ethanol washes, as described previously. After 3 rinses with 1× PBS, slides were covered with prehybridization buffer containing denatured salmon sperm DNA and incubate at 37°C for 1 hr. After 3 rinses with 1× PBS, 250 μg of the biotin labeled HPV16 DNA was then added in hybridzation buffer to the slides and incubated at 37°C overnight. Slides were washed 3 times with 1× PBS and developed with Tyramide amplification and DAKO reagent as described previously. Hematoxalin and eosin staining was performed for improved contrast.

### 2.8. Statistical Analyses

A power analysis was performed to determine if significance would be achieved at the chosen sample size. For the purpose of this study, 200 samples were chosen; 100 HIV-positive and 100 HIV-negative. At that sample size, and an alpha set at 0.5 and degrees of freedom set at 2, the distinguishable effect size was 0.3, which is well within the allowable range for this study. For the purpose of analyzing a difference in HPV genotype distributions as a function of HIV status, the Kruskall-Wallis non-parametric test was used to detect differences in the medians between groups. Alternatively, Fisher’s Exact Test was used.

## 3. Results

Our study samples were comprised of paraffin embedded placental tissues which were obtained from HIV-positive and HIV-negative women who had been admitted to the University Teaching Hospital (UTH), Lusaka, Zambia for delivery of their babies. These samples were then shipped to Nebraska Center for Virology (NCV) and stored in the Tissue Bank of Dr. Charles Wood, who kindly provided them for our HPV analyses. We chose to analyze these samples in order to determine if HPV status was influenced by HIV. Very little is currently known about HPV infection in the placenta or the effect of HIV on those infections. Two-hundred samples, (100 HIV-negative and 100 HIV-positive) were analyzed (Table 1).

### 3.1. Detection of HPV in Placental Tissues

HIV and HPV are endemic in Zambia. The prevalence rates for HPV as high as 97.2% have been previously reported among HIV-positive Zambian women [28]. Based on recent studies that suggest that HPV can infect epithelial linings of the placenta [29] [30], we decided to probe for the presence of HPV DNA in placental tissues of both HIV-positive and HIV-negative Zambian women. This goal of this study was to determine the effect of HIV upon HPV infection and pathogenesis in the placenta. For this purpose, we used a cohort of 200 placental tissue samples from which we extracted genomic DNA and PCR amplified HPV DNAs using GP5+/6+ and CPI/II primers. These redundant primers can amplify up to 40 different types of HPVs. The status of the cellular DNA in the samples was monitored by *β*-Actin PCR and any samples that did not test positive for *β*-Actin PCR were excluded from the study. We excluded 4 samples of the 100 HIV-negative samples because of poor cellular DNA recovery. To make up for this, we added 4 HIV-positive samples. This left us with 96 HIV-negative samples and 104 HIV-positive genomic DNA samples for HPV analysis.

### 3.2. Determination of HPV Prevalence

The overall HPV prevalence rate was 85.0%, with the HIV-negative group accounting for 86(43.0%), and the HIV-positive group accounting for 84(42.0%). There was no statistically significant effect of HIV upon HPV prevalence overall (Fischer’s Exact test: p > 0.5; p = 0.112).

### 3.3. HPV Genotype Distribution

**Figure 2:**
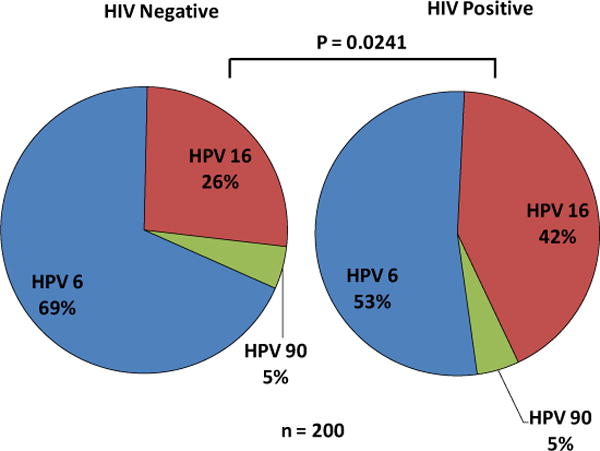
The distribution of HPV in HIV-negative and HIV-positive placental samples. Three types of HPVs were isolated including the low risk (LR)-HPV6, high-risk (HR)-HPV16 and the rarely reported HPV90. The pie-chart on the left side shows the distribution of HPV genotypes in HIV-negative placental tissues and; The pie-chart on the right side shows the distribution of HPV genotypes in HIV-positive placental tissues. To determine the HPV genotypes, the PCR products were cloned into the pGEMT vector and sequenced. There was a significant difference (p < 0.05; p = 0.0241) in the distribution of HPV16 between the HIV−/HPV16+ and the HIV+/HPV16+ groups, with an odds ratio of 2.1. There was no significant difference (p > 0.05; p = 0.0864) in the distribution of HPV6 between the HIV−/HPV16+ and the HIV+/HPV16+ groups.

Cloned PCR products were sequenced and the HPV genotypes determined by BLAST search against the NCBI database. Three HPV genotypes were identified: Low-Risk (LR) HPV6, the High-Risk (HR) HPV16 and the rarely reported HPV90. Eighty-six (89.6%) HIV-negative samples tested positive for HPV and 83(96.5%) of these were genotyped whereas the HPV genotype status of the remaining 3(3.5%) samples could not be determined. The HIV-negative group harbored HPV90 which accounted for 4(5.0%) whereas the LR-HPV6 and HR-HPV16 accounted for 57(69.0%), and 22(26.0), respectively (Figure 2).

Of the 84(80.8%) HIV-positive samples that tested positive for HPV, 83(98.8%) were genotyped while the HPV genotype status of 1(1.2%) sample could not be determined. The HPV positive group had the same HPV90 distribution 4(5.0%) as the HIV-negative group. However, the HPV6 distribution in this group was 44(53%) and that of HPV16 was 35(42%). Comparison of the HPV16 distribution between the HIV+ (42%) and the HIV− (26%) groups using Fisher’s exact test showed a statistically significant difference (p < 0.05; p = 0.0241) with a 2.1 odds ratio. We did not find a significant difference (p > 0.05; p = 0.0864) in the distribution of HPV6 between the two groups.

### 3.4. Socio-Demographic Characteristics of Women by HPV Status and Genotype Distribution

We wanted to know whether the socio-demographic characteristics of women in our study were associated with prevalence of particular HPV genotypes found in placental tissues.

Table 1 shows the socio-demographic characteristics of the women by HPV status and genotype distribution and HIV status. The data shows that overall; the most significant effects were in HIV-dependent effects on HPV genotype distribution. We found a significant difference (Fischer’s Exact test: p < 0.05; p = 0.0241) in HPV16 genotype distribution between the HIV+ and HIV− women. There was no significant difference in HPV6 (Fischer’s Exact test: p > 0.05; p = 0.0864) distribution between the two groups.

Further comparison of age, years of education and household size between the HIV-positive and HIV-negative women did not reveal any significant effects on HPV genotype distribution (Fishers’ Exact test: p > 0.05).

### 3.5. Comparison of Resident HPV Genotypes in the Vaginal and Placental Compartments

Comparison of the vaginal lavage HPV types to those that we discovered in the placental compartment showed that the HPV genotypes resident in the two compartments were different, except for sample 919 which had a concordance of HPV6 in both compartments (Table 2). We also noted that the vaginal compartment had mixed HPV infections while the placental compartment had single infections. Furthermore, we noted that infections were often exclusive to a single compartment (10/15 samples). The results of the analysis of HPV genotypes in the vaginal versus the placental compartments suggest that tissue compartment specific distributions of HPVs exist within individuals.

### 3.6. Detection of HPV16 L1 Protein

The productive infection by human papillomavirus is characterized by the expression of the late capsid protein (L1) which by itself can assemble into virus like particles (VLPs) [31] [32]. Our PCR results showed that both HIV-positive and HIV-negative placental tissues were positive for HPV16 by PCR of the L1 region. We therefore sought to determine whether L1 protein expression could be detected in placental trophoblasts. For this purpose we used a Mouse monoclonal anti-HPV16 L1 (H16.V5) antibody [33].

**Figure 3:**
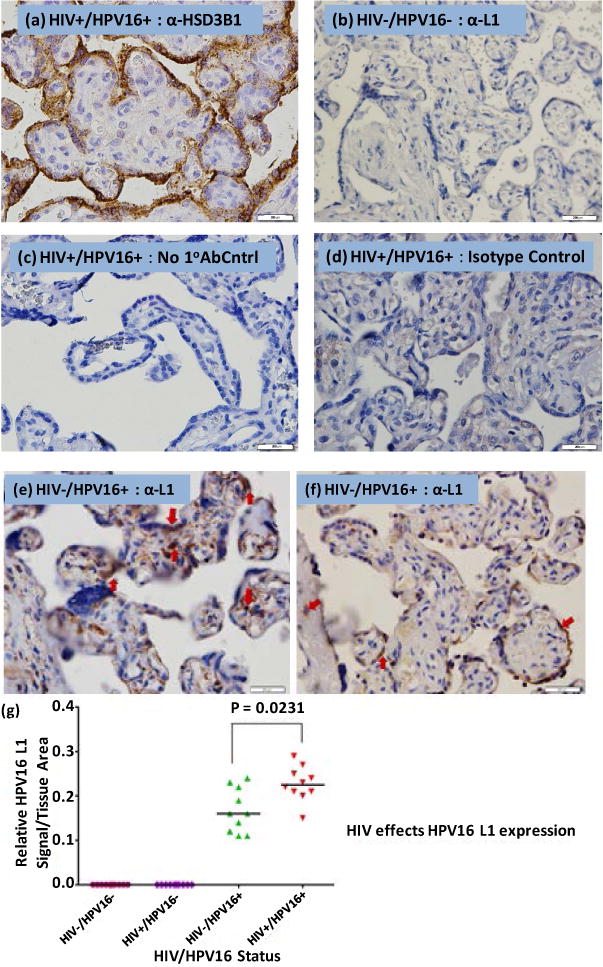
HPV16 L1 immunohistochemistry staining. (a) HSD3B1 trophoblast marker; (b) Placental trophoblasts stained with a Mouse monoclonal anti-IgG isotype control; (c) Placental trophoblasts stained without a primary antibody; (d) Placental trophoblasts of an HIV−/HPV− tissue stained with anti-V5L1 antibody; (e) Placental trophoblasts of an HIV+/HPV16+ tissue stained with anti-V5L1 antibody; (f) Placental trophoblasts of an HIV−/HPV16+ tissue stained with anti-V5L1 antibody; (g) Quantification of HPV16 L1 protein in HIV−/HPV16+ and HIV+/HPV16+ sectioned placental tissues. Image Pro-Premier offline 9.0 was used to determine the HPV16 L1 relative levels in HIV−/HPV16+ and HIV+/HPV16+ sectioned placental tissues. The HPV16 L1 protein levels in all groups were determined in duplicate (results not shown). The average HPV16 L1 values, normalized to the tissue areas, were compared using Kruskal-Wallis test. The bars represent the medians of the HPV16 L1 signal. The median HPV16 L1 signal varied significantly (p < 0.05; p = 0.0001) across all the groups. The expression of the L1 protein was significantly different (p < 0.05; p = 0.0231) between the HIV−/HPV16+ and HIV+/HPV16+ groups. We observed an HIV dependent effect on the expression of the L1 protein. As expected, all the HIV−/HPV16− and HIV+/HPV16− samples were negative for HPV16 L1 staining. Slides were counterstained with haematoxylin and eosin and pictures were taken for 0.5 seconds with an Olympus digital camera linked to the microscope. The bar on the right bottom indicates 200 μM in scale.

The trophoblast marker (Figure 3(a)) served as a reference for the location of the syncytiotrophoblast cells. To control for background staining due to non-specific binding, we used normal serum derived IgG4 isotype antibody. As expected, we did not observe any background staining (Figure 3(b)). Using an HIV−/HPV+ placental tissue, we also stained for L1 in the absence of the primary antibody and again, observed no detectable background signal (Figure 3(b)). There was no detectable L1 signal in the HIV−/HPV− placental tissue (Figure 3(d)).

The HPV16 L1 protein was detected in HIV+/HPV16+ and HIV−/HPV16+ placental trophoblasts (Figure 3(e) and Figure 3(f)). Both cytotrophoblasts and syncytiotrophoblasts stained positive for the L1 protein, with most of the staining occurring along columns of syncytiotrophoblasts which are the terminally differentiated trophoblast cells. The L1 protein expression was also observed in the decidual cells of the maternal side of the placenta.

It has been hypothesized that the HIV-1 tat protein can transactivate the Long Control Region (LCR) of the HPV genome and upregulate the expression of E6 and E7 genes. Therefore, we chose to assess whether HIV could have an indirect effect on HPV16 L1 expression. We quantified the relative expression of L1 protein present in placental tissue samples (Figure 3(g)). To achieve this, we used Image-Pro Premier Offline 9.0 software, which we trained to discriminate between background signal and the actual HPV16 L1 signal. Subtraction of background signal gave the relative L1 signal per tissue area. The HPV16 L1 protein levels in all groups were determined in duplicate and the average L1 values, normalized to the tissue areas, were compared statistically. The median HPV16 L1 signal varied significantly (**Kruskal Wallis:** p < 0.05; p = 0.0001) across all the groups. The expression of the L1 protein was significantly different (p < 0.05; p = 0.0231) between the HIV−/HPV16+ and HIV+/HPV16+ groups. We concluded that expression of the L1 protein was clearly influenced by HIV status.

**Figure 4:**
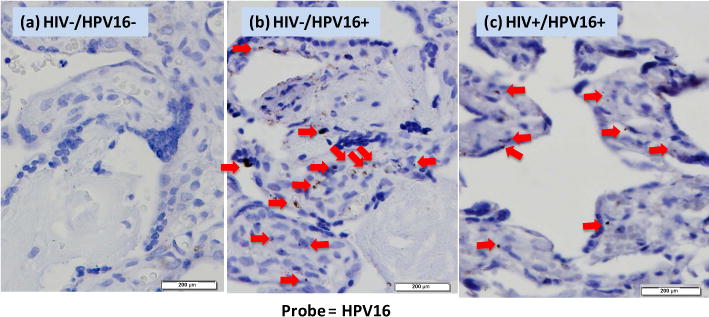
HPV16 *in situ* hybridization in placental trophoblasts. Tissues from HIV−/HPV16−, HIV−/HPV16+, HIV+/HPV16+ were hybridized with full-length HPV16 DNA and detected with tyramide amplification system and DAB for colorization. The red arrows indicate the hybridization signals. Slides were counterstained with haematoxylin and eosin and pictures were taken for 0.5 seconds with an Olympus digital camera linked to the microscope. The bar on the right bottom indicates 200 μM in scale.

We performed *in situ* hybridization to test for evidence or HPV DNA with trophoblast cells of the placenta. We found clear evidence of HPV16 DNA present in the nuclei of trophoblast cells of the placenta either from HIV-negative or HIV-positive patients (Figure 4). HPV negative samples showed no cross-reaction with the HPV16 DNA probe. Overall, these data are consistent with HPV infection in the placenta.

## 4. Discussion

Recent studies have demonstrated that HPV can infect epithelial trophoblast cells of the placenta [12] and HPV DNA has also been detected by PCR in placentas obtained trans-abdominally from women undergoing amniocentesis [8]. These and other studies led us to explore the prevalence of HPV and the effect of HIV on HPV genotype distribution within the placental compartment.

To the best of our knowledge, we are the first group to investigate HPV infection of the placenta in the context of HIV infection. Using redundant primer sets, we were able to detect HPV DNA in both HIV-negative and HIV-positive placental tissues.

Married HIV-negative women were found to be placenta positive for HPV [73/81(90.1%)] as were married HIV-positive women [75/95(78.9%)] as shown in Table 1. The high incidence of HPV infections among HIV+ and HIV− married women in this population could be explained in part by multiple sexual partners that they may have had by the time they were married as most of them are between 15 and 25 years. In low income countries such as Zambia, young women engage in sex earlier than do women in more affluent countries. Furthermore, there is a greater rate of women engaging in sex for monetary benefit, at a young age, which puts them at greater risk of contracting STDs, such as HPV and HIV. Placental samples were HPV6 positive at high rates, HIV+ (53%) and HIV− (69%), yet there was no significant difference (p > 0.05) between the two groups (Figure 2). We did not observe any significant differences between married HIV+ and HIV− women in most socio demographic status (Years of education and Household size) as a function of HPV genotype. Nevertheless, we observed a significant difference (Fishers’ Exact test: p < 0.05; p < 0.0001; 2-tailed) in HIV-positive women aged 15 – 25 and 26 – 36 years [OR 0.0057, 95% CI, 0.000323 – 0.0992] when age was used as function of testing positive for HPV. HIV+ women aged 26 – 36 years were more at risk of testing positive for HPV than those aged 15 – 25 years. One plausible explanation for this observation is that women in this age group are most likely to have had multiple sexual partners, which puts them at risk of being infected with both HIV and HPV.

The HPV prevalence rate has widely been reported to be at least 80% [34]. HPV prevalence rates as high as 97.2% have been previously reported among HIV-positive Zambian women [28]. In our study, we found that the prevalence of HPV in the HIV-positive placental samples 84/104(80.8%) was slightly lower than that of the HIV-negative ones 86/96(89.6%), differing from previous studies [15] [16] [18]. The difference in HPV prevalence between the HIV-positive and HIV-negative placental tissues was not significant (Fisher’s exact test; p > 0.05; p = 0.112). In assessing the effect of HIV on HPV genotype distribution in the placental compartment, we found only three different types of HPV, namely the low risk HPV6, the high risk HPV16 and the rarely isolated HPV90 (Figure 2). We discovered that the incidence of the high risk HPV16 in HIV-positive placental tissues was greater than that in HIV-negative tissues. There was a significant difference (p < 0.05; p = 0.0241) in the incidence of HPV16 between the two groups of placental tissues (Figure 2). We found that HIV-positive placental tissues were two times more likely to harbor the high risk HPV16 than the HIV-negative placental tissues. This result was in agreement with that obtained in a study conducted by Ng’andwe and colleagues in which they found a nine-fold increase in the incidence of the high risk HPV18 in HIV-positive versus HIV-negative vaginal lavage samples of Zambian women. It appears that the replication efficiency of the high risk HPVs is increased in patients whose immune system is compromised [34].

Interestingly, our placental HPV genotyping distribution differed from the vaginal lavage results that Ng’andwe *et al.* had previously obtained from the same patients. This observation suggests that different HPVs could prefer different compartments (Table 2). In the previous vaginal lavage study by Ng’andwe and colleagues in our laboratory on the same patient samples [24] HPV16 and HPV18 were recovered in high abundance. This is in contrast to our present placental study in which HPV6 and 16 were the main genotypes isolated. Whereas in our study of the vaginal HPV distribution, we recovered 20 different HPV genotypes, the placental compartment was limited to only 3, suggesting very selective growth conditions in that tissue. Arguably, HPV6 appears to be the most successful at exploiting the placental compartment. It is important to acknowledge that differences in the rate of PCR detection of HPVs in different compartments could influence these results. The analysis of HPV in the vaginal and placental compartments was performed under exactly the same conditions using the same protocols. Second round of PCR for samples that tested negative for HPV was used to ensure that we did not leave out HPV positive samples.

Although HPV90 has been previously reported in an underserved population in United States, there is dearth of information to determine its prevalence, distribution and disease association [35]. The presence of HPV90 in cervical lesions as previously reported and now in placental tissues of our study samples may imply that this genotype has the potential to replicate in various epithelial compartments. To the best of our knowledge, we are the first group to report the presence of HPV90 in Zambian specimens and this finding suggests that unique HPV isolates may be present in this population.

In vitro studies have shown that the human papillomavirus can replicate productively in placental trophoblasts. In our study, we performed immunohistochemistry staining for the HPV16 L1 protein (Figure 3) using a monoclonal anti-HPV16 V5 L1 antibody [27] on patient-derived placental samples. The L1 protein is the major viral capsid protein that is expressed during the late phase of the HPV life cycle and can by itself spontaneously assemble into virus like particles [31] [32]. Neither the HIV−/HPV− negative control (Figure 3(d)) nor did the anti-IgG4 isotype control (Figure 3(b)) gave a positive signal for HPV16 L1. Additionally, the HIV+/HPV16+ tissue section stained without anti-L1 (Figure 3(c)) gave no detectable signal for HPV16 L1. We observed positive staining for L1 protein expression in HIV+/HPV16+ (Figure 3(e)) and HIV−/HPV16+ (Figure 3(f)) placental trophoblasts. The staining was observed both in cytropohoblast and cyncytiotrophoblast cells. It appears that HPV16 establishes its productive infection in these cells. To further elucidate this assertion, we wanted to perform electron Microscopy on the placental samples so that we could show the presence of HPV16 virion particles in these cells. This was however not possible because the membranes were disrupted during paraffin embedding of the placental tissues. Therefore as an alternative to this method, we intend to show evidence of the HPV16E1^E4 splicing product in trophoblastic cells in our follow up study. Having identified the cells that were positive for HPV16 L1 staining, we quantified the relative HPV16 L1 protein using Pro-Premier Offline 9.0. We observed that the relative HPV16 L1 median signal varied significantly (Kruskal-Wallis: p < 0.05; p = 0.0001) across all the 4 groups (Figure 3(g)). The elevated expression of HPV16+ L1 in HIV+/HPV16+ trophoblastic cells in comparison to the HIV−/HPV16+ suggests that HIV has an effect on HPV16 L1 protein expression. To further explore this, we compared the HPV16 L1 relative signal between the HIV+/HPV16+ and HIV−/HPV16+ tissue samples (Figure 3(g)) and observed that there was a significant difference (Mann-Whitney: p < 0.05; p = 0.0231). The HIV dependent effect on HPV16 L1 expression in placental trophoblasts could be attributed to the ability of HIV-1 *tat* to transactivate the HPV LCR [15] [36]. Thus, our HP16 L1 immunohistochemistry result corroborates the in vitro report that HPV can replicate productively in 3A trophoblasts in tissue culture [37].

Infection of the cervix with the HR-HPVs such as HPV16 and 18 are associated with cervical dysplasia [38] [39]. Interestingly, we did not find clear evidence of pathology in the placenta related to the presence of HR-HPV16. One plausible explanation for the absence of pathology in trophoblastic cells is that extravillous trophoblast cells express neither Major Histocompatibility Complex (MHC) class I nor class II molecules [40] [41]. The lack of MHC classes I and II expression implies that the HPV virus can freely replicate without inducing an immune response.

Finally, in this study, we have shown the presence of HPV in placental trophoblasts using both polymerase chain reaction and HPV16 L1 immunohistochemistry methods. We have also shown, for the first time, the effect of HIV on HPV infection in placental trophoblasts.

We also found evidence of HPV16 in placental trophoblast cells by *in situ* hybridization. Further corroboration of HPV infection of placental trophoblastic cells is needed. Finally, our main focus for this study was to determine the effect of HIV on high risk HPV16 infection of placental trophoblasts. We therefore now intend to perform immunohistochemistry analysis for the low risk HPV6 and compare the results with those of HPV16 IHC. Overall, our results support the conclusion that a subset of HPVs infects the placenta and their prevalence is influenced by HIV status.

## Figures and Tables

**Table 1:** Socio-demographic characteristics of women by HPV status and genotype distribution and HIV status.

	HPV−		HPV+		HPV16		HPV6		HPV90	

	HIV−	HIV+	HIV−	HIV+	HIV−	HIV+	HIV−	HIV+	HIV−	HIV+

Total	n = 10 (10.8%)	n = 20 (19.4%)	n = 83 (89.2%)	n = 83 (80.6%)	n = 22 (26.5%)	n = 33 (39.8%)	n = 57 (68.7%)	n = 44 (53.0%)	n = 4 (4.9%)	n = 4 (4.8%)
**Marital Status**										
Married	8(9.9%)	20(21.1%)	73(90.1%)	75(78.9%)	18(22.2%)	29(50.0%)	51(63.0%)	42(42.0%)	4(4.9%)	4(4.8%)
Single	2(18.2%)	0(0.0%)	9(81.8%)	7(100.0%)	4(36.4%)	5(71.4.0%)	5(45.5%)	2(28.6%)	0(0.0%)	0(0.0%)
Widowed	0(0.0%)	0(0.0%)	1(100%)	1(100%)	0(0.0%)	1(100%)	1(100%)	0(0.0%)	0(0.0%)	0(0.0%)
**Age (years)**										
15 – 25	10(10.8%)	20(58.8%)	83(89.2%)	14(41.2%)	22(2.5%)	14(100%)	57(68.7%)	0(0.0%)	4(4.8%)	0(0.0%)
26 – 36	0(0.0%)	0(0.0%)	0(0.0%)	62(100.0%)	0(0.0%0	19(30.6%)	0(0.0%)	43(69.4%)	0(0.0%)	0(0.0%)
37 – 47	0(0.0%)	0(0.0%)	0(0.0%)	7(100.0%)	0(0.0%0	0(0.0%0	0(0.0%)	1(20.0%)	0(0.0%)	4(80.0%)
**Years of Education**										
None	0(0.0%)	2(28.6%)	3(60.0%)	5(71.4%)	0(0.0%)	2(28.6%)	3(60.0%)	3(42.9%)	0(0.0%)	0(0.0%)
1 to 7	4(8.3%)	8(19.5%)	44(91.7%)	34(82.9%)	13(27.1%)	21(51.2%)	30(62.5%)	13(31.7%)	1(2.1%)	0(0.0%)
8 to 12	4(11.1%)	11(33.3%)	32(88.9%)	33(75.0%)	6(16.7%)	6(13.6%)	24(66.7%)	25(56.8%)	2(5.6%)	2(4.5%)
>12	0(0.0%)	1(8.3%)	6(100.0%)	11(91.7%)	2(33.3%)	6(50.0%)	3(50.0%)	3(250%)	1(16.7%)	2(16.7%)
**Household Size**										
1 to 3	5(11.4%)	7(18.9%)	39(88.6%)	30(81.8%)	15(34.1%)	14(37.8%)	21(47.7%)	15(40.5%)	3(6.8%)	1(2.7%)
4 to 6	2(6.9%)	7(16.7%)	27(93.1%)	35(28.3%)	6(20.7%)	12(28.6%)	21(72.4%)	23(54.8%)	0(0.0%)	0(0.0%)
7 to 9	2(18.2%)	3(17.6%)	9(81.8%)	14(82.4%)	0(0.0%)	5(35.7%)	9(81.8%)	6(35.3%)	0(0.0%)	3(17.6%)
10 to 12	1(12.5%)	3(42.9%)	7(87.5%)	4(57.1%)	1(12.5%)	4(57.1%)	6(75.0%)	0(0.0%)	0(0.0%)	0(0.0%)

**Table 2:** HPV distribution in the vagina and placental compartments.

		Compartment	

Sample ID	HIV Status	Vagina	Placenta
79	−	None	6
112	−	None	90
132	−	None	None
919	−	6	6
78	−	None	6
186	−	81/62	6
1542	−	None	None
1543	−	None	None
107	+	45	6
126	+	None	6
64	+	None	16
81	+	16	None
133	+	51	None
184	+	83	None
984	+	53/6	None
